# Optimization of Xylanase Production from* Aspergillus foetidus *in Soybean Residue

**DOI:** 10.1155/2018/6597017

**Published:** 2018-04-11

**Authors:** Luana Cunha, Raquel Martarello, Paula Monteiro de Souza, Marcela Medeiros de Freitas, Kleber Vanio Gomes Barros, Edivaldo Ximenes Ferreira Filho, Mauricio Homem-de-Mello, Pérola Oliveira Magalhães

**Affiliations:** ^1^Laboratory of Natural Products, School of Health Sciences, University of Brasília, Asa Norte, 70910900 Brasília, DF, Brazil; ^2^Laboratory of Enzymology, Department of Cell Biology, University of Brasília, Asa Norte, 70910900 Brasília, DF, Brazil

## Abstract

Enzymatic hydrolysis is an important but expensive step in the process to obtain enzyme derived products. Thus, the production of efficient enzymes is of great interest for this biotechnological application. The production of xylanase by* Aspergillus foetidus* in soybean residues was optimized using 2 × 2^3^ factorial designs. The experimental data was fitted into a polynomial model for xylanase activity. Statistical analyses of the results showed that variables pH and the interaction of pH and temperature had influenced the production of xylanase, with the best xylanase production level (13.98 U/mL) occurring at fermentation for 168 hours, pH 7.0, 28°C, and 120 rpm.

## 1. Introduction

Xylanases (EC 3.2.1.8) are found in both fungi and bacteria. They randomly catalyze the endohydrolysis of 1,4-*β*-D-xylosidic linkages in xylan [1]. According to the CAZy database (http://www.cazy.org), xylanases are classified under glycosyl hydrolase (GH) families 5, 7, 8, 9, 10, 11, 12, 16, 26, 30, 43, 44, 51, and 62 based on their amino acid sequences and structures [2].

Family 10 consists of endo-1,4-*β*-xylanases, endo-1,3-*β*-xylanases, and cellobiohydrolases. The major enzymes of this family are endo-1,4-*β*-xylanases; however, substrate specificity studies revealed that these may not be entirely specific for xylan and may not be active on low molecular mass cellulose substrates. In effect, it has been found that the replacement of one or two xylose residues by glucose is normally tolerated by the xylanases of this family, with this generally resulting in a lowered catalytic efficiency [2].

In contrast to other families, family 11 is monospecific, it consists solely of xylanases. Moreover, these xylanases are “true xylanases” as they are exclusively active on D-xylose containing substrates. They have a lower catalytic versatility than family 10 xylanases and indeed the products of their action can be further hydrolyzed by the family 10 enzymes. These xylanases are characterized by a high pI, low molecular weight, and a *β*-sheet structure [2].

Supplementary studies (data not shown) revealed a low percentage of *α*-helix (~3–6%) and high percentage of *β*-sheets (~43–48%). This suggests xylanase from* Aspergillus foetidus* is classified as a member of family 11. These results are in accordance with literature, in which xylanases present approximately 3–5% of *α*-helix structures and a higher percentage of *β*-sheets [3, 4].

Among the many microbial sources, filamentous fungi are especially interesting as they secrete these enzymes into the medium and their xylanase levels are very much higher than those found in yeasts and bacteria [5]. Several xylanases have been reported from these fungal strains for various industrial and biotechnological applications. In order to fulfill specific industrial needs, enzymes must possess pH stability, thermostability, high specific activity, and most importantly high affinity for the substrate [6, 7].

Xylanases are extensively used in the paper and pulp industry, as well as in baking, animal feed, biofuels production, fruit and vegetable processing, manufacture of bread, food, and drinks, textiles, xylitol production, saccharification of agricultural, and industrial and municipal wastes among other utilities [5, 8]. The successful industrial application of xylanase requires its cost-effective production in bulk quantity. The production cost can be reduced by optimizing the fermentation medium and the process, for example, using cheap agroresidue as carbon source [7, 9].

Brazil is the second biggest producer of soybean worldwide, harvesting 96.5 kton in 2016, just behind United States, with 106.9 kton in 2016 [10, 11]. Therefore, soybean residues represent the major byproduct of processing soybean industry [12], which could be used as a carbon source for the production of enzymes [13].

Thus, the experimental design statistical approach for enzyme production using a response surface methodology (RSM) is an alternative strategy to reduce the production cost. Recently, RSM has been utilized successfully to improve product yield and to reduce development time and cost of biotechnological processes [7].

In this work, we employed Central Composite Design (CCD) for the planned statistical optimization of xylanase activity of an* Aspergillus foetidus* strain isolated from Brazilian Savannah, grown in submerged fermentation, using soybean residue as substrate.

## 2. Materials and Methods

### 2.1. Soybean Residue Pretreatment

Soybean residue was autoclaved at 121°C for 2 hours and thoroughly washed with tap water. After autoclaving, it was dried at 65°C for 48 hours and then grounded to form a relatively homogeneous blend. A fine powder was obtained and used as a substrate for xylanase production [13].

### 2.2. Organism and Enzyme Production

The fungi* Aspergillus foetidus* was obtained from the microorganisms' collection of Laboratory of Enzymology from University of Brasília. The* Aspergillus foetidus* was kindly provided by Professor Dr. Edivaldo Ximenes, Depositary Microorganisms Center: Collection of microorganisms for phytopathogens and weeds control from Embrapa Genetic Resources, accredited by Genetic Heritage Management Council by CGEN deliberation n° 67 published in the DOU in 13.09.2004, Section 1, page 53, linked to the project “Biotechnological processes in application of hollocelulases from filamentous fungi”, Process n° 010237/2015-1, University of Brasília. It is maintained in potato dextrose agar (PDA) medium at −80°C.

The spore concentration was determined by counting under a microscope with a Newbauer chamber and adjusted with sterile saline solution (0.9%) to a final concentration of 1 × 10^7^ spores/mL.

For xylanase production, an aliquot (1 mL) of spore suspension (10^7^ spores/mL) was inoculated into 250 mL Erlenmeyer flasks containing 50 mL of liquid medium (0.4% peptone, 0.4% yeast extract, 0.2% KH_2_PO_4_, 0.8% NaH_2_PO_4_, 0.25% MgSO_4_) at pH 7.0 with 2% (w/v) of soybean residue. The cultures were incubated at 28°C with constant agitation at 120 rpm for 7 days.

After the culture growth, the medium was filtered through a Büchner funnel with filter paper (Whatman n°1) and stored at −20°C. The resulting filtrate, here called crude extract, was used as a source of xylanase.

### 2.3. Enzyme Assay

The xylanase activity was determined by mixing 50 *μ*L of enzyme solution with 100 *μ*L of birchwood xylan (10 mg/mL) in 50 mM sodium acetate buffer, pH 5.4 at 50°C for 30 min [14]. The release of the reducing sugar was measured using the DNS method [15]. The absorbance was read at 540 nm by spectrophotometry (Shimadzu UV-1800) and the xylanase activity was expressed as *μ*mol of reducing sugar released per min per milliliter (IU/mL). Xylose was used as standard.

Protein concentration was measured by the method of Bradford, using bovine serum albumin as standard [16].

### 2.4. Experimental Design and Statistical Analysis

To analyze the effects of the agitation (*X*_1_), temperature (*X*_2_), and pH (*X*_3_) at enzymatic production of xylanase in medium with soybean residue, two factorial designs were employed (FD1 and FD2). For both, a 2^3^ factorial design with three center points and axial points was employed (Table 1). The factors were coded to allow the analysis of variance (ANOVA) by response of enzymatic activity (*Y*).

Center points were defined based on previous methodology used in our laboratory (data not shown); axial, −1, and +1 points were determined in order to evaluate significant differences. FD2 was planned aiming to increase the difference observed.

The Design-Expert® software, version 9.0.6.2, was used for regression and graphical analysis of the data. Only the factors with significance higher than or equal to 5% (*p* < 0.05) were considered.

For each factorial design, 17 experiments (determination of xylanase activity) were performed and are shown in results section.

## 3. Results and Discussion

### 3.1. Enzyme Induction

**Figure 1:**
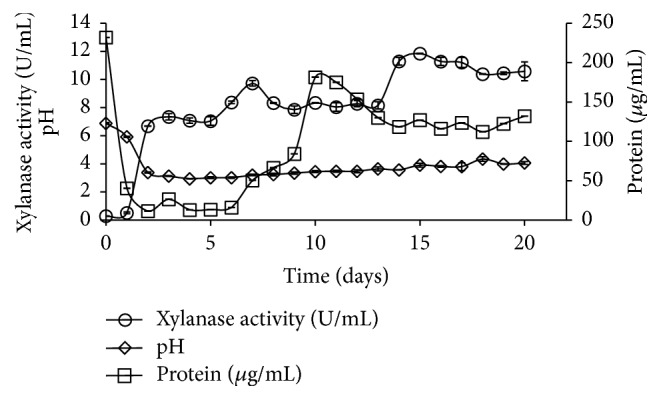
Time course of xylanase produced by* A. foetidus.*

To optimize induction time,* Aspergillus foetidus* xylanase specific activity was assessed every day, for 20 days (Figure 1). The specific activity was determined during this period.

The highest xylanase activity was after 15 days of culture (11.84 U/mL). On the 7th day of culture, the xylanase activity was 9.72 U/mL, and specific activity had its maximum value, which was 810.41 U/mg during the period of analysis. A specific activity of purified* Aspergillus foetidus* xylanase was 1196.53 U/mg (data not shown) [4].

The specific activity is an important parameter to assess the enzymatic activity in relation to the amount of proteins in the sample. In general, high specific activity represents the highest level of target enzyme. For this reason, the 7th day was chosen for this study.

The xylanase specific activity from* Aspergillus foetidus *cultivated with soybean residue is consistent with previous reports, which xylanases from* Aspergillus sp.* cultivated in different residues also present high activity levels, as described below.

Delabona et al. (2013) found a specific xylanolytic activity for* Aspergillus fumigatus*, 1055.6 U/g and 558.3 U/g in wheat bran and soybean, respectively, after 5 days of solid state fermentation. In the same work, he found for* Aspergillus niger,* a specific xylanolytic activity of 1285.0 U/g, 484.2 U/g, and 1050.0 U/g using as residue wheat bran, soybean, and wheat bran with sugarcane bagasse, respectively [17]. Supplementing it, Yang et al. (2015) found for* Aspergillus fumigatus *submerged liquid culture with sugarcane bagasse a xylanolytic activity of 53.1 U/mg [18]; and Farinas et al. (2010) after 3 days of* Aspergillus niger* solid state fermentation found for xylanase activity 13.24 U/mL [19].

The amount of protein oscillated during the culture period, suggesting this result may include other enzymes besides xylanase, which are concomitantly produced and also participate in the substrate degradation process. The induction profile followed the pH variation, with maximum value of 5.92 on the 1st day of culture and minimum value of 2.94 on the 4th day. Seventh day pH was 3.20. This result indicates a xylanase production in acidic medium.

### 3.2. Factorial Design

The activity of the xylanase present in the crude extract produced by filamentous fungus* Aspergillus foetidus* grown on soybean residue under submerged liquid culture was assessed. Variation on agitation, temperature, and pH effects on xylanase activity were evaluated using the statistical design of experiments and RSM analysis.  Table 2 presents the results of the complete factorial design for xylanase activities under the different conditions evaluated. Tables 3 and 4 exhibit the coefficients of the mathematical model and statistical parameters.

#### 3.2.1. Xylanase FD1

In the first study, the independent variables pH, pH^2^, and pH *∗* temperature (*C*, *C*^2^, *BC*) had a significant effect on the xylanase production (*p* < 0.05). Interactions between agitation and temperature (*AB*) and interactions between agitation and pH (*AC*) had no significance (*p* > 0.05). Under the levels tested in the factorial design, the variables agitation and temperature did not interact with each other. Subsequently, the xylanase activity is not significantly modified.

**Figure 2:**
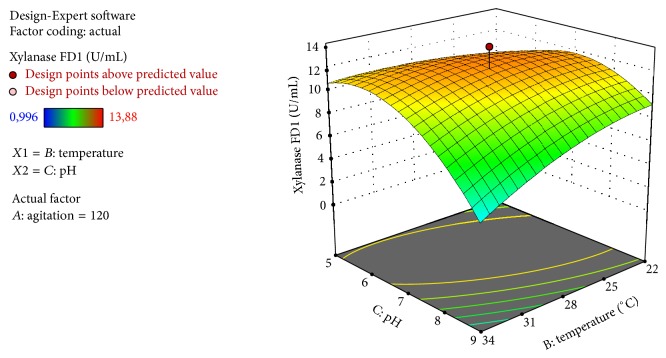
Response surface for xylanase FD1 as a function of the variables temperature and pH.

Data were fitted to a quadratic model with three central points. The statistical significance of the equation was checked and the determination coefficient (*R*^2^) was calculated to be 0.88, indicating that 88% of the variability in the response could be explained by the model. In addition, the *F* test (5.87 times higher than the listed *F* value at 90% level of confidence) was satisfactory for the prediction of the model used to describe response surface plot of the enzyme activity as a function of pH and temperature (Figure 2). Higher experimental value of enzymatic activity was found at the condition of central point, which is at 120 rpm, pH 7, and 28°C.

Other studies of xylanase report optimum pH, temperature, and agitation at specific values. De Souza Moreira et al. (2013) found an optimum pH and temperature of pH 6.0, 50°C at 120 rpm and pH 5.0, 45°C at 120 rpm for xylanases produced by* A. terreus* under submerged fermentation. Ang et al. (2013) found a maximum activity at 60°C and optimum pH of 4.0 for xylanase produced by* A. fumigatus *under solid state fermentation (SSF) [20]. The advantage of using the statistical methodology was the definition of an optimum temperature and pH range, rather than a specific value, allowing more flexibility during process development [19].

The lack-of-fit test did not result in a significant *p* value, indicating that the model is sufficiently accurate to predict the factors responses within the ranges studied. The “lack-of-fit *F*-value” of 1.38 implies that the lack of fit is not significant relative to the pure error. There is a 47% (*p* = 0.47) chance that a “lack of-fit *F*-value” this large could occur due to noise.

The “Pred *R*-Squared” of 0.242 is not as close to the “Adj *R*-Squared” of 0.732 as one might normally expect (difference is more than 0.2). This may indicate a large block effect or a possible problem with the model. “Adeq Precision” measures the signal-to-noise ratio. A ratio of 7.91 indicates an adequate signal (a ratio greater than 4 is desirable). This model can be used to navigate the design space.

The response equation obtained is the following: *Y*_1_ = −81.111 + 0.431*∗A* + 1.784*∗B* + 12.937*∗C* + 0.006*∗A∗B* + 0.009*∗A∗C* − 0.153*∗B∗C* − 0.003*∗A*^2^ − 0.027*∗B*^2^ − 0.748*∗C*^2^, where *Y*_1_ is the predicted xylanase activity in U/mL; *A*, *B*, and *C* are the coded variables of agitation, temperature, and pH, respectively. The equation in terms of actual factors can be used to make predictions about the response for given levels of each factor.

The negative effect of the factors means that an increase in one of them will reduce the enzymatic activity. For xylanase activity, pH showed a positive effect while temperature *∗* pH and pH^2^ showed a negative effect, within the range evaluated. Furthermore, the significance of the interaction effect between pH and temperature revealed synergistic effect of these variables; that is, the variables pH and temperature well-adjusted could modify the xylanase activity. The pH effect was higher than the temperature effect, as can be verified for the coefficient values listed in Table 3. A similar result was found by Farinas et al. (2010) and Singh et al. (2009) on optimization of parameters for cellulase and xylanase from* Aspergillus niger* and cellulase from* Aspergillus heteromorphus*, respectively. The authors found that the change in temperature was less importante than changes in pH, within the range evaluated. As the pH varies, the charge of the substrate and ionic components of the substrate changes, affecting the activity of the enzymes [19, 21].

#### 3.2.2. Xylanase FD2

In the second study of xylanase, the range of agitation, pH, and temperature were expanded in relation to the range of the previous study, in order to evaluate a possible positive effect in the xylanase activity.

The computed *F*-value of 4.39 implies the model is significant at a high confidence level (Table 4). The probability *p* value was also relatively low (*p* < 0.05), indicating the significance of the model. The coefficient of variation (*R*^2^ = 0.84) indicates a high correlation between the experimentally observed and predicted values and indicates the degree of precision with which the treatments are compared.

The “lack-of-fit *F*-value” of 3.27 implies the lack of fit is not significantly relative to the pure error. There is a 25.04% (*p* = 0.2504) chance that a “lack-of-fit *F*-value” this large could occur due to noise. Additionally, “Adeq Precision” of 5.88 indicates an adequate signal. This model can be used to represent the design space.

The independent variable pH, pH^2^, and temperature^2^ (*C*, *C*^2^, *B*^2^) had a significant effect on the xylanase production (Table 4). The other correlations had no significance (*p* > 0.05).

The response equation obtained for the second model was as follows: *Y*_2_ = −54.322 + 0.113*∗A* + 1.997*∗B* + 10.506*∗C* + 0.000*∗A∗B* − 0.001*∗A∗C* − 0.056*∗B∗C* − 0.000*∗A*^2^ − 0.028*∗B*^2^ − 0.713*∗C*^2^, where *Y*_2_ is the predicted xylanase activity and *A*, *B*, and *C* are the coded variables of agitation, temperature, and pH, respectively. The equation in terms of actual factors can be used to make predictions about the response for given levels of each factor.

**Figure 3:**
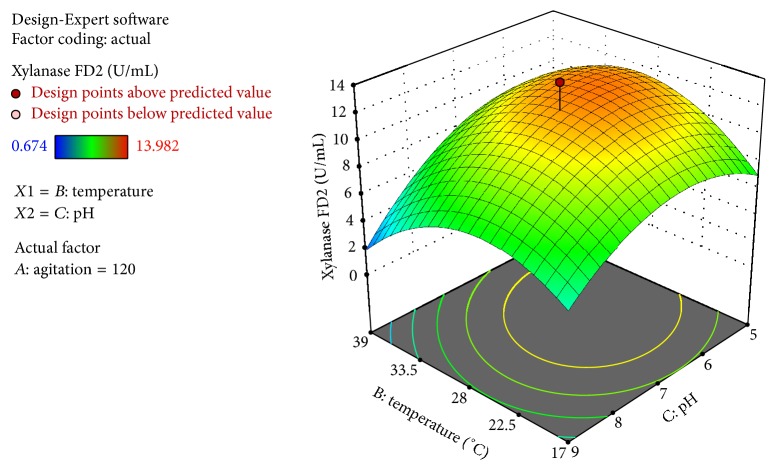
Response surface for xylanase FD2 as a function of the variables temperature and pH.

To investigate the effects of pH and pH *∗* temperature on xylanase production, the three-dimensional contour plot was used to assess the effects of pH and temperature on xlylanase production (Figure 3). Highest xylanase activity in this second experiment was 13.98 U/mL (120 rpm, 28°C, pH 7.0). This activity was similar to the one achieved in the first study, which shows that the increase in the interval between levels of the variable did not influence the optimal condition observed in the first design. The increase of the interval, however, was able to demonstrate that the pH was the variable with greater influence in the tested design.

Xylanase activities for the first and second models were 12.028 U/mL and 11.989 U/mL, respectively, for the optimal conditions of the model (120 rpm, 28°C, and pH 7). There were very small differences in the predicted response and the activity observed during the experiments, confirming that the results are in accordance of the RSM study. The xylanase from* A. foetidus* is more effective in comparison to xylanase from* A. niger*,* A. flavus*, and* A. fumigatus* as can be seen when you compare the obtained results to some data from the literature.

Guimaraes et al. (2013) found a xylanolitic activity for* Aspergillus niger* and* Aspergillus flavus* of 10.50 and 11.92 U/mL, respectively, using as residue wheat bran 0.5% and corncob 0.5%. Supplementing it, Naseeb et al. (2015) found for* A. fumigatus* pea peel substrate under Solid State Fermetation (SSF) 13.97 U/mL [22, 23].

## 4. Conclusions

In this study, we investigated the feasibility of filamentous fungus* Aspergillus foetidus *to produce high level of xylanase enzyme in a liquid medium. This study highlighted a newly isolated strain* A. foetidus* which could produce xylanase in soybean residue medium, which is cheap and abundant. The best conditions of xylanase production were pH 7.0, 120 rpm, and 28°C (168 hours). In future studies, statistical optimization of medium, physical factors, and scaling up studies in bioreactor should be used as an alternative to contribute toward the economics of biotechnological processes.

## Figures and Tables

**Table 1:** Code and level of factors chosen for the trials for xylanase FD1 and FD2.

Independent variable	Symbol	Range and level				
Xylanase FD1		−1.68	−1	0	+1	+1.68
Agitation (rpm)	*X*_1a_	86	100	120	140	154
Temperature (°C)	*X*_2a_	18	22	28	34	38
Ph	*X*_3a_	3.6	5	7	9	10.3

Xylanase FD2						
Agitation (rpm)	*X*_1b_	60	84	120	156	180
Temperature (°C)	*X*_2b_	10	17	28	39	46
pH	*X*_3b_	3.6	5	7	9	10.3

**Table 2:** Results obtained for 2^3^ factorial design with parameters: values of pH, temperature, and agitation.

Run order	Coded levels			Xylanase FD1 (U/mL)	Xylanase FD2 (U/mL)
	*X*_1_	*X*_2_	*X*_3_		
**1**	−1	−1	−1	8.965	8.423
2	+1	−1	−1	8.525	8.730
3	−1	+1	−1	9.955	11.243
4	+1	+1	−1	10.603	8.512
5	−1	−1	+1	8.979	5.737
6	+1	−1	+1	8.378	3.004
7	−1	+1	+1	0.996	0.806
8	+1	+1	+1	4.783	0.674
9	0	0	0	10.811	13.982
10	0	0	0	13.880	11.506
11	0	0	0	11.709	10.915
12	0	0	−1.68	3.864	3.976
13	0	0	+1.68	1.407	1.358
14	0	−1.68	0	8.571	0.705
15	0	+1.68	0	8.131	1.016
16	−1.68	0	0	7.731	9.480
17	+1.68	0	0	8.447	8.349

**Table 3:** Analysis of variance (ANOVA) for the model regression 1.

Source	SS	DF	MS	*F-value*	*p value*
Model ^1^	167.53	9	18.61	5.87	0.0147
*A*, agitation	1.55	1	1.55	0.49	0.5073
*B*, Temp.	6.27	1	6.27	1.98	0.2027
*C*, pH	26.56	1	26.56	8.37	0.0232
*AB*	3.75	1	3.75	1.18	0.3130
*AC*	1.11	1	1.11	0.35	0.5730
*BC*	26.81	1	26.81	8.45	0.0227
*A* ^2^	12.81	1	12.81	4.04	0.0844
*B* ^2^	10.68	1	10.68	3.37	0.1092
*C* ^2^	101.05	1	101.05	31.86	0.0008
Residual	22.20	7	3.17		
Lack of fit	17.22	5	3.44	1.38	0.4700
Pure error	4.98	2	2.49		
Total	189.73	16			

^1^Model regression is xylanase activity = −81.111 + 0.431*∗A* + 1.784*∗B* + 12.937*∗C* + 0.006*∗A∗B* + 0.009*∗A∗C* − 0.153*∗B∗C* − 0.003*∗A*^2^ − 0.027*∗B*^2^ − 0.748*∗C*^2^; *R*^2^ = 0.8830; SS, sum of squares; DF, degrees of freedom; MS, mean square. Significance level = 95%.

**Table 4:** Analysis of variance (ANOVA) for the model regression 2.

Source	SS	DF	MS	*F-value*	*p value*
Model ^2^	274.80	9	30.53	4.39	0.0319
*A*, agitation	3.79	1	3.79	0.55	0.4844
*B*, Temp.	1.25	1	1.25	0.18	0.6839
*C*, pH	70.78	1	70.78	10.19	0.0152
*AB*	0.024	1	0.024	0.007	0.9549
*AC*	0.024	1	0.024	0.007	0.9545
*BC*	12.16	1	12.16	1.75	0.2274
*A* ^2^	4.67	1	4.67	0.67	0.4393
*B* ^2^	137.41	1	137.41	19.78	0.0030
*C* ^2^	91.73	1	91.73	13.20	0.0084
Residual	48.63	7	6.95		
Lack of fit	43.34	5	8.67	3.27	0.2504
Pure error	5.30	2	2.65		
Total	323.43	16			

^2^Model regression is xylanase activity = −54.322 + 0.113*∗A* + 1.997*∗B* + 10.506*∗C* + 0.000*∗A∗B* − 0.001*∗A∗C* − 0.0560*∗B∗C* − 0.000*∗A*^2^ − 0.028*∗B*^2^ − 0.713*∗C*^2^; *R*^2^ = 0.849; SS, sum of squares; DF, degrees of freedom; MS, mean square. Significance level = 95%.
